# Examining downstream effects of concizumab in hemophilia A with a mathematical modeling approach

**DOI:** 10.1016/j.jtha.2024.10.028

**Published:** 2024-11-12

**Authors:** Kenji Miyazawa, Alan E. Mast, Adam R. Wufsus, Michael Dockal, Marianne Kjalke, Karin Leiderman

**Affiliations:** 1 Quantitative Biosciences and Engineering Program, Colorado School of Mines, Golden, Colorado, USA; 2 Thrombosis and Hemostasis Program, Versiti Blood Research Institute, Milwaukee, Wisconsin, USA; 3 Rare Disease, Novo Nordisk, Inc., Plainsboro, NJ, USA; 4 Rare Blood Disorders, Novo Nordisk A/S, Novo Nordisk Park, Maaloev, Denmark; 5 Department of Mathematics, University of North Carolina at Chapel Hill, Chapel Hill, NC; 6 Computational Medicine Program, University of North Carolina at Chapel Hill, Chapel Hill, North Carolina, USA

**Keywords:** concizumab, hemophilia A, mathematical modeling, TFPI-antibody

## Abstract

**Background:**

Tissue factor (TF) pathway inhibitor (TFPI) is an anticoagulant protein that inhibits factor (F)Xa, the TF-FVIIa-FXa complex, and early forms of the prothrombinase complex. Concizumab is a monoclonal antibody that blocks FXa inhibition by TFPI and reduces bleeding in hemophilia.

**Objectives:**

To examine how concizumab impacts various reactions of TFPI to restore thrombin generation in hemophilia A using mathematical models.

**Methods:**

A compartment model was used to estimate plasma concentrations of free concizumab and its complexes with TFPIα and TFPIβ. Concizumab was integrated into a flow-mediated mathematical model of coagulation, and a small injury was simulated under hemophilia A conditions. Simulations were then analyzed to determine how concizumab’s blockade of TFPI anticoagulant activities, specifically the inhibition of FXa in plasma and on platelets, inhibition of TF:FVIIa at the subendothelium, and prior sequestration of plasma TFPIα to the endothelium via TFPIβ, altered thrombin generation.

**Results:**

Concizumab improved simulated thrombin generation in hemophilia A by simultaneously altering all 3 mechanisms of the TFPI anticoagulant blockade examined. Concizumab sequestered ~75% of plasma TFPIα through the formation of ternary TFPIα-concizumab-TFPIβ-complexes. For all TF levels, reducing the TFPIα plasma concentration had the largest impact on the lag time, followed by blocking TFPIα inhibition of TF:FVIIa:FXa and subsequently by blocking TFPIα inhibition of FXa in plasma and on the platelet surface.

**Conclusion:**

The effectiveness of concizumab is mediated through the blockade of TFPI anticoagulant activities in plasma and on multiple physiological surfaces. An important and previously unrecognized function of concizumab was the sequestration of plasma TFPIα to the endothelium.

## 1 | INTRODUCTION

Hemophilia A is a genetic bleeding disorder characterized by a deficiency in coagulation factor (F)VIII due to the defect in the *F8* gene, resulting in impaired thrombin generation via the propagation phase of coagulation and, consequently, lack of fibrin-stabilization of the initial platelet clots sealing a vascular injury. The severity of hemophilia is classified by the plasma activity of FVIII: severe if <1%, moderate if between 1% and 5 %, and mild if between 5% and 40% of the reference interval (50% to 150%) [1]. Patients with severe hemophilia may receive FVIII concentrates intravenously several times per week, but even with intensive prophylactic therapy, low FVIII trough levels may result in suboptimal bleed protection and arthropathy [2–4]. Gene therapy is an alternative treatment that also decreases bleeding frequency [5]. However, the limitations of gene therapy include the durability of FVIII expression [6,7].

Hemophilia is also treated using antibodies that mimic activated FVIII or block the activity of endogenous coagulation inhibitors. Concizumab is a humanized monoclonal antibody against tissue factor (TF) pathway inhibitor (TFPI) that is intended for subcutaneous prophylactic treatment for persons with hemophilia A or B with or without inhibitory alloantibodies to FVIII or FIX [8–10]. The efficacy of concizumab has been verified through *in vivo* and *ex vivo* experiments during phase 1 studies, where thrombin generation was greatly accelerated in a dose-dependent manner in blood from people with hemophilia. The safety and efficacy of concizumab in hemophilia prophylaxis have been assessed in phase 2 and 3 clinical trials [9,10].

Concizumab targets the Kunitz 2 (K2) domain of TFPI, which binds to and inhibits activated coagulation FXa [11–13]. FX is activated in the initiation phase of coagulation by the TF-activated FVII (FVIIa) complex formed when subvascular TF is exposed to blood upon vascular injury. There are 2 alternatively spliced isoforms of TFPI in humans: TFPIα, a soluble protein in plasma, in platelets, and in the extracellular matrix, and TFPIβ, a glycosylphosphatidyl inositol anchored protein on endothelial cell surfaces. TFPIα is released into plasma from platelets upon activation. TFPIα inhibits the initiation phase of coagulation by simultaneous inhibition of TF:FVIIa and FXa immediately after FX is activated by TF:FVIIa [14]. Plasma TFPIα also binds to FXa and partially B-domain cleaved FV, the cofactor for FXa in the prothrombinase complex, on the surface of activated platelets. The B-domain in this FV form is cleaved by FXa [15], resulting in FV-“half” (FV-h) that retains a sequence with hydrophobic amino acids followed by an acidic region of the B-domain that tightly binds to a stretch of basic amino acids in the C-terminus of TFPIα. Thrombin cleavage of FV-h releases the entire B-domain to produce FVa that does not bind to TFPIα. TFPIα, therefore, reduces the activity of early forms of prothrombinase (FXa:FV-h) by slowing prothrombinase assembly, ie, by blocking the generation of FXa via the TF:FVIIa complex and by inhibiting prothrombinase assembled with FV-h [16–18]. Since concizumab blocks the interaction between TFPI and the active site of FXa, it reduces TFPI anticoagulant activity toward FXa, TF:FVIIa [12], and early prothrombinase. However, the relative importance of these 3 mechanisms for the overall effect of concizumab is not known. Furthermore, TFPIα and TFPIβ are expressed in distinct locations within the vasculature and extracellular matrix, where they exert differential inhibitory activity toward FXa, TF:FVIIa, and early prothrombinase [17]. In this study, we used mathematical models to systematically analyze the impact of concizumab on TFPI’s individual anticoagulant mechanisms and, thereby, define their relative importance for restoring thrombin generation in hemophilia.

## 2 | METHODS

### 2.1 | Compartment model

**FIGURE 1:**
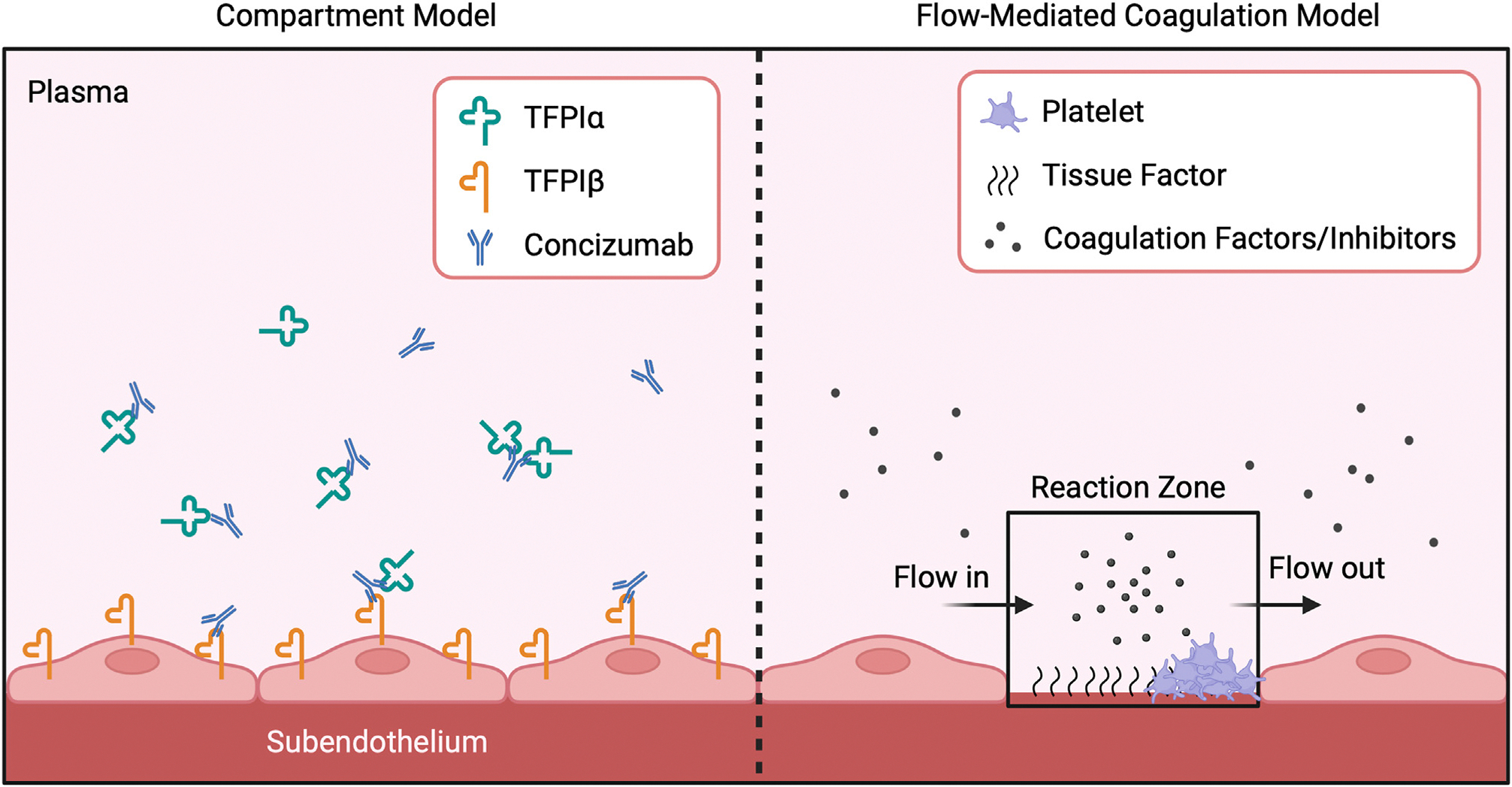
Model schematics. The compartment model (left) predicts levels of concizumab bound to tissue factor pathway inhibitor (TFPI)α and TFPIβ. Output from the compartment model is input to the flow-mediated coagulation model (right), which simulates thrombin generation that results from reactions occurring in a small reaction zone above the exposed tissue factor and subjected to flow.

We developed a compartment model (Figure 1, left; network diagram, Supplementary Figure S1) to simulate blood coagulation, assuming well-mixed reactions (Table), including concizumab binding to TFPIα in plasma and concizumab binding to TFPIβ on endothelial cells. Concizumab, with 2 binding sites for the K2 domain of TFPI on each molecule [12,20], can bind to 1 or 2 TFPIα molecules and to 1 TFPIα and 1 TFPIβ molecule simultaneously, so we also included a reaction for the formation of the ternary complex TFPIα:C:TFPIβ at the endothelium. The notation “C” represents the concizumab species within the mathematical model. We translated these reactions into ordinary differential equations using the law of mass action and numerically simulated them through time until a steady state was reached. The system of ordinary differential equations was solved numerically using the odeint function from the SciPy library in Python. We defined steady state to mean that the numerical solutions for each model species (eg, TFPIα, C, C:TFPIα, etc.) had negligible changes for at least 5 minutes.

We set the total plasma TFPIα and endothelial TFPIβ concentrations to 2.5 nM and 18 nM, respectively. We defined the total plasma concizumab concentration to be the sum of free plasma concizumab and concizumab bound to 1 or 2 TFPIα molecules and set this to be 4 nM, close to the 4.4 nM, which was the geometric mean plasma concentration in a phase 3 clinical trial where patients received a steady-state dose between 0.15 and 0.25 mg/kg daily [10]. We defined the total intravascular concizumab concentration as the amount of concizumab administered into the bloodstream, which includes plasma and cellular surface-bound forms. The effects of metabolism, degradation, and clearance of concizumab:TFPI (C:TFPI) complexes on the total intravascular concizumab concentration were neglected in this model.

### 2.2 | Flow-mediated coagulation model

The mathematical model in this study is an extension of our previously published model [21] and now includes reactions involving concizumab and TFPIα. These reactions are described in the next sections and listed in the Table. Below, we give a brief summary of the flow model, but further details can be found in our previous work [21,22]. Here, we used the model to simulate how steady-state concentrations of concizumab, plasma TFPIα, and concizumab-bound TFPIα, estimated from the compartment model in the presence and absence of TFPIβ, influenced thrombin generation upon plasma exposure to TF.

The flow-mediated coagulation model simulates TF-initiated coagulation reactions occurring in a reaction zone (RZ) situated just above a small injury (10 × 10 μm) that exposes the subendothelial surface (Figure 1, right). All reactants and intermediates are assumed to be well-mixed, which means that changes in space are neglected, and only concentration changes over time are considered. The influence of flow and diffusion to and from the RZ is considered with a simplified, single rate of mass transfer. The model includes 3 different types of platelets: quiescent platelets in plasma, activated platelets attached to the subendothelium, and activated platelets attached to other platelets. Platelets are activated when there is sufficient thrombin (>1 nM) and when they attach to the subendothelium through von Willebrand factor and collagen. Platelets attached to the subendothelium are assumed to cover the RZ and decrease TF activity there. Activated platelets activate quiescent platelets at a constant rate that loosely represents the release of agonists, eg, adenosine diphosphate. Biochemical coagulation reactions occur on the subendothelium, in plasma, and on activated platelet surfaces.

Model input requires initial concentrations for all model species (proteins and platelets) in both the plasma and RZ, which are set to the normal levels found in healthy human plasma (Supplementary Table S2). The initial concentrations of free plasma concizumab, plasma TFPIα, and concizumab-bound TFPIα are based on the values estimated from the compartment model (steady state values reported in Supplementary Table S1). The plasma concentration of TFPIα and its complexes were reduced to 20% of their values estimated from the compartment model. This was done because approximately 20% of plasma TFPIα exists in the free, full-length form, while the remaining portion is C-terminal truncated and bound to lipoproteins [23]. It is important to note that while lipoprotein-bound TFPI has no effect on the initiation of coagulation in clotting assays initiated with low TF concentration (diluted prothrombin time assay) [24], it does reduce peak thrombin in TF-initiated thrombin generation assays, indicating that this pool of plasma TFPI alters propagation of coagulation *in vitro*. Nevertheless, its function *in vivo* remains unclear [25] and is therefore neglected here.

### 2.3 | Concizumab binding K2 domain of TFPIα

The model includes reactions where concizumab can bind to free TFPIα in the plasma, to TFPIα:FV-h in plasma or bound to platelet surfaces, and to TFPIα bound to prothrombinase via FV-h (reactions 4, 6, 8, and 10 in the Table). As mentioned above, concizumab can bind 2 TFPIα molecules, and we assume this occurs in 2 steps whereby the C:TFPIα complex binds an additional free TFPIα and forms TFPIα:C:TFPIα (reaction 5 in the Table).

### 2.4 | C:TFPIα complex binding FV-h

Another assumption based on the flexibility of TFPIα is that its C-terminus is available to bind FV-h, even when the K2 domain is bound to concizumab. Thus, the model included reactions where the C:TFPIα complex binds FV-h both in plasma and bound to the platelet surface (reactions 7 and 9 in the Table).

### 2.4 | Example equations

Biochemical reactions were translated into ordinary differential equations based on the law of mass action. For example, the following equation describes the rate of change of concizumab in the compartment model, which depends on concizumab binding to TFPIα and TFPIβ:

$$\frac{dC}{dt}=-k_{\alpha }^{+}C\cdot TFPI\alpha -k_{\beta }^{+}C\cdot \;TFPI\beta +k_{\alpha }^{-}[C:\;TFPI\alpha ]+k_{\beta }^{-}[C:\;TFPI\beta ].$$

The rate of change is balanced with 2 negative terms (loss of concizumab due to its binding with TFPIα and TFPIβ) and 2 positive terms (concizumab becoming free after its dissociation from TFPIα and TFPIβ). Binding and unbinding rates are indicated by superscripts + and −, respectively. Species connected with a colon in a bracket represent a complex, eg, (C:TFPIα) represents the complex of concizumab bound to TFPIα. Note that the equation for concizumab in the coagulation model (vs the compartment model) will contain more terms on the right-hand side of the equation that account for the reactions in the Table. See Supplementary material for the list of equations. The total system of ordinary differential equations was solved numerically using a Fortranbased solver, interfaced with Python for efficient computation.

## 3 | RESULTS

### 3.1 | Compartment model estimated plasma levels of concizumab and its complexes with TFPI

**FIGURE 2:**
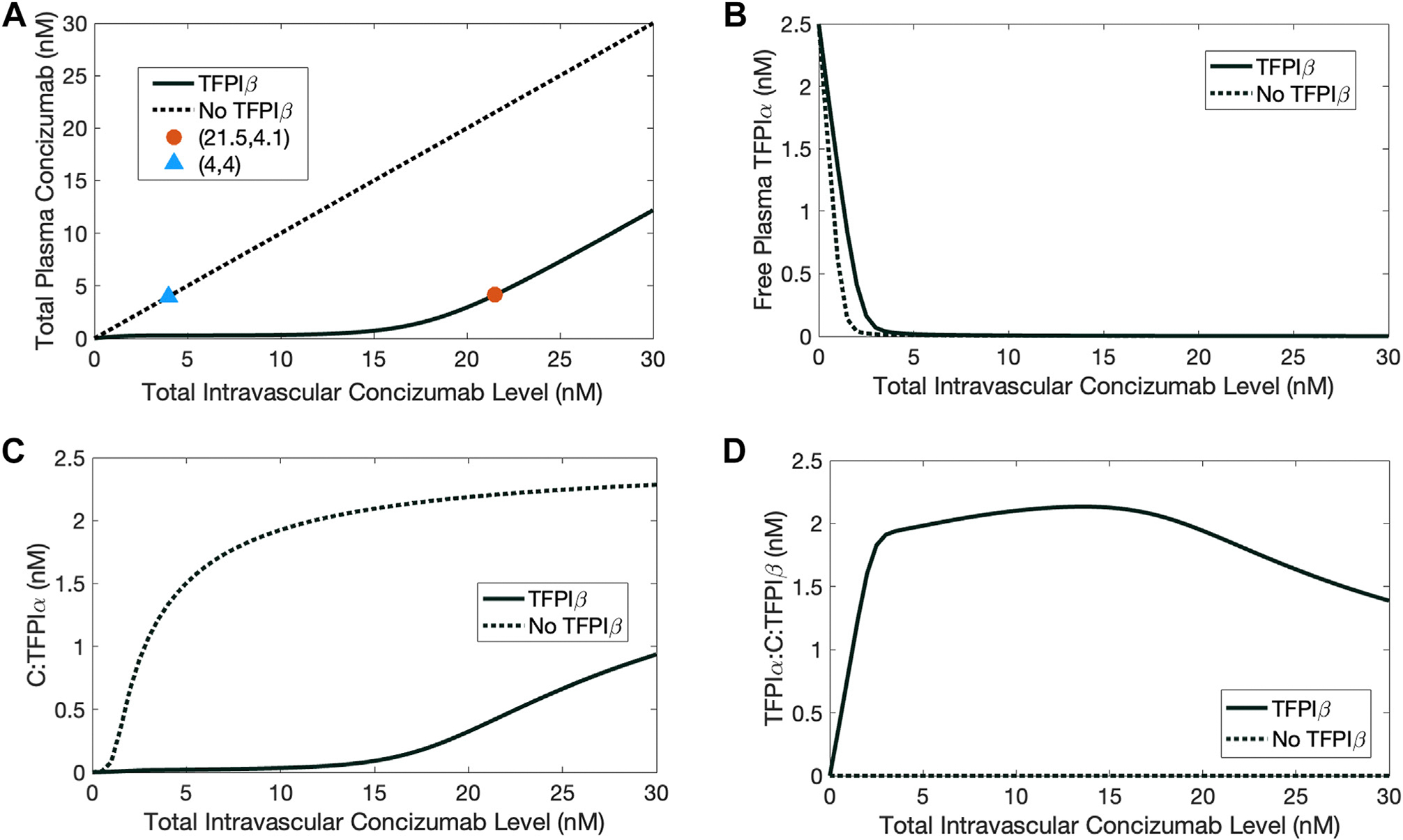
Selected steady-state concentrations estimated with the compartment model and plotted as functions of total intravascular concentrations of concizumab (C). Simulations are shown with tissue factor pathway inhibitor (TFPI)β (solid black lines) and without TFPIβ (dotted black lines). Total plasma C concentrations are shown (A) with an orange dot and blue triangle indicating the total intravascular concizumab level leading to 4 nM total plasma concizumab. Steady-state concentrations of free plasma TFPIα (not bound to C), C:TFPIα, and TFPIα:C:TFPIβ are shown in (B–D), respectively.

The geometric mean of the plasma concizumab concentration observed in clinical studies is approximately 4 nM [10]. However, the proportions of free concizumab and C:TFPI complexes in the plasma are unknown. The compartment model was used to estimate these proportions. In the model, the total plasma concizumab concentration was defined as the sum of 3 components: free plasma concizumab, concizumab bound with 1 TFPIα molecule (C:TFPIα), and concizumab bound to 2 TFPIα molecules (TFPIα:C:TFPIα). Steady-state total plasma concizumab concentrations were computed for a range of initial intravascular concizumab concentrations (0–30 nM). Because concizumab binds to TFPIβ on the endothelium, the initial intravascular concizumab concentrations that produced 4 nM plasma concizumab were 21.5 nM when TFPIβ was present and 4 nM when it was absent (Figure 2A). Steady-state free TFPIα was >95% depleted when total intravascular concizumab concentrations exceeded 4 nM (Figure 2B), but its partitioning into complexes differed if TFPIβ was present or absent (Figure 2C, D). When TFPIβ was present, 15.52 nM concizumab was bound to TFPIβ only, 1.85 nM formed a ternary complex with TFPIα and TFPIβ, and 0.55 nM was bound to plasma TFPIα only (Supplementary Table S1). Thus, concizumab sequestered 74% of the plasma TFPIα to the endothelium via the formation of TFPIα:C:TFPIβ complexes. In the absence of TFPIβ, TFPIα remained in plasma but bound to concizumab. These trends held for all intravascular concizumab concentrations. In summary, the compartment model estimated the plasma and endothelial proportions of concizumab corresponding to a clinically relevant plasma concentration of concizumab. These estimates identified a procoagulant role for concizumab, including sequestration of TFPIα from the plasma onto the endothelium.

### 3.2 | Effect of concizumab on flow-mediated thrombin generation in hemophilia A

The effects of concizumab on thrombin generation in hemophilia A were characterized using our mathematical model of flow-mediated coagulation [21,22]. Thrombin curves generated from this model look different than curves from static coagulation assays, eg, calibrated automated thrombograms. The major difference is that in static coagulation, reactions are limited by the concentration of reactants and inhibitors present, so thrombin generation curves rise and fall. Under flow, reactants are constantly being supplied and flow away, so sustained thrombin generation is possible.

**FIGURE 3:**
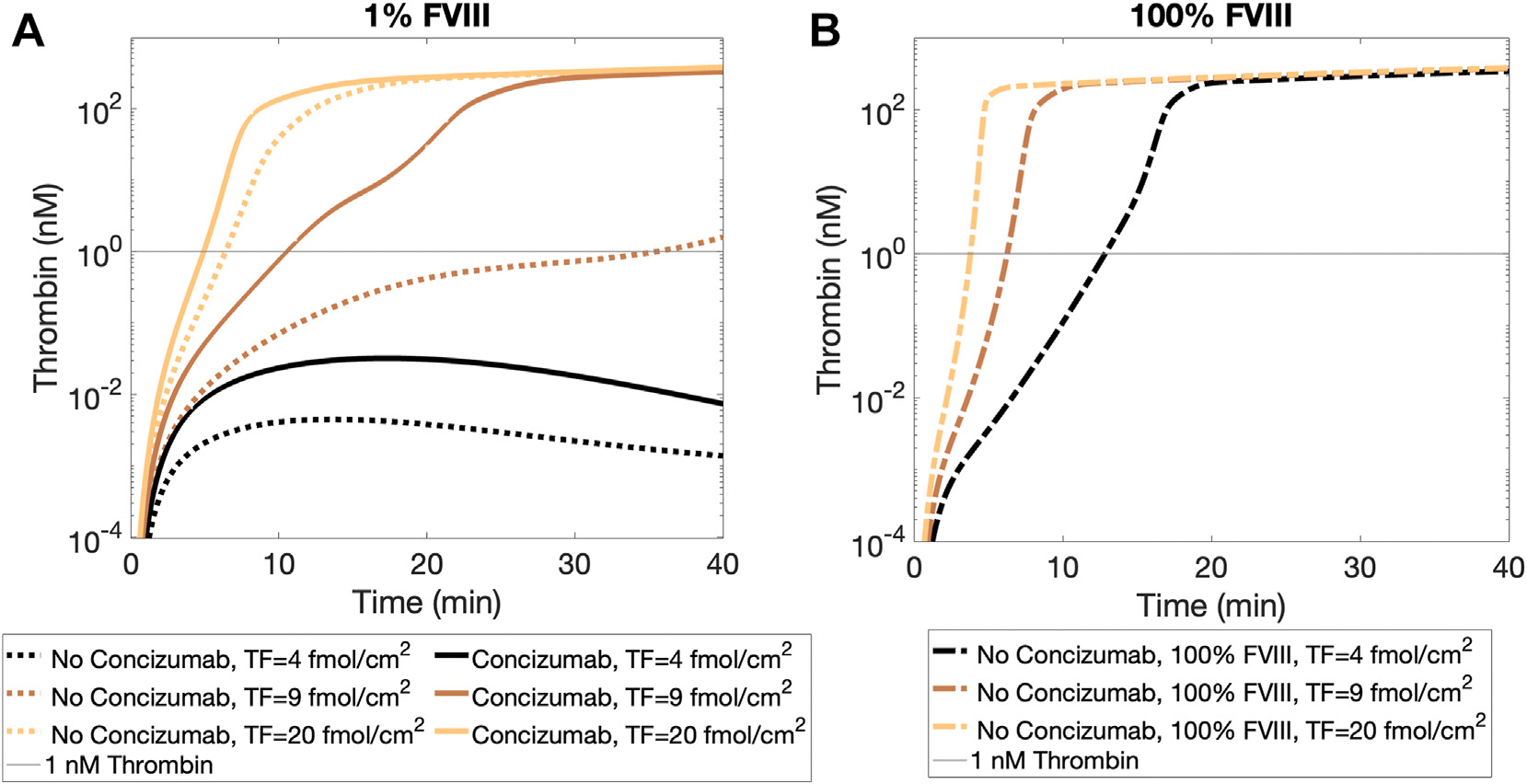
Thrombin generation time courses with varied tissue factor TF, concizumab, and factor (F)VIII. TF was low (black, 4 fmol/cm^2^), intermediate (brown, 9 fmol/cm^2^), or high (copper, 20 fmol/cm^2^). Simulations were run in the presence (solid curves) and absence (dotted and dash-dotted) of concizumab. FVIII is set to 1% of normal to simulate hemophilia A (A) or to 100% of normal (B). The thin black line represents 1 nM thrombin. The lag time was defined as the time when the thrombin curves crossed this line.

TF densities of 4, 9, and 20 fmol/cm^2^ were used to represent weak (<1 nM thrombin within 40 minutes), intermediate (35 minutes lag time), and strong (7 minutes lag time) thrombin generation without concizumab (Figure 3, left panel). The lag time is defined here as the time at which the simulated thrombin generation reaches 1 nM concentration. The effects of steady-state concentrations from the compartment model (Supplementary Table S1) for concizumab and its complexes with TFPIα on thrombin generation were examined. When hemophilia A was simulated by reducing FVIII to 1% (from 1 nM at 100% to 0.01 nM at 1%, as used in our previous work [26]), concizumab did not elevate thrombin generation above 1 nM within 40 minutes when TF was low (4 fmol/cm^2^). However, concizumab shortened the lag time for 1 nM thrombin generation from 35.6 minutes to 10.6 minutes at the intermediate TF level (9 fmol/cm^2^) and from 6.3 minutes to 5 minutes at the high TF level (20 fmol/cm^2^). In all cases where thrombin surpassed 1 nM, concentrations of thrombin reached between 200 and 300 nM by 40 minutes. In comparison, at normal FVIII level (Figure 3, right panel) and in the absence of concizumab, the lag times were 13 minutes, 7 minutes, and 3 minutes, respectively, at these TF densities. Thus, in our flow-mediated model, concizumab had the greatest impact at the intermediate TF density. At low TF density, the inhibition of TFPIα by concizumab was inadequate to prompt coagulation. Conversely, when TF density was high, such that the lag time was under 10 minutes without concizumab, concizumab enhanced thrombin generation, but its effects were less pronounced.

### 3.3 | Concizumab effects on coagulation biochemistry during flow-mediated thrombin generation in hemophilia A

**FIGURE 4:**
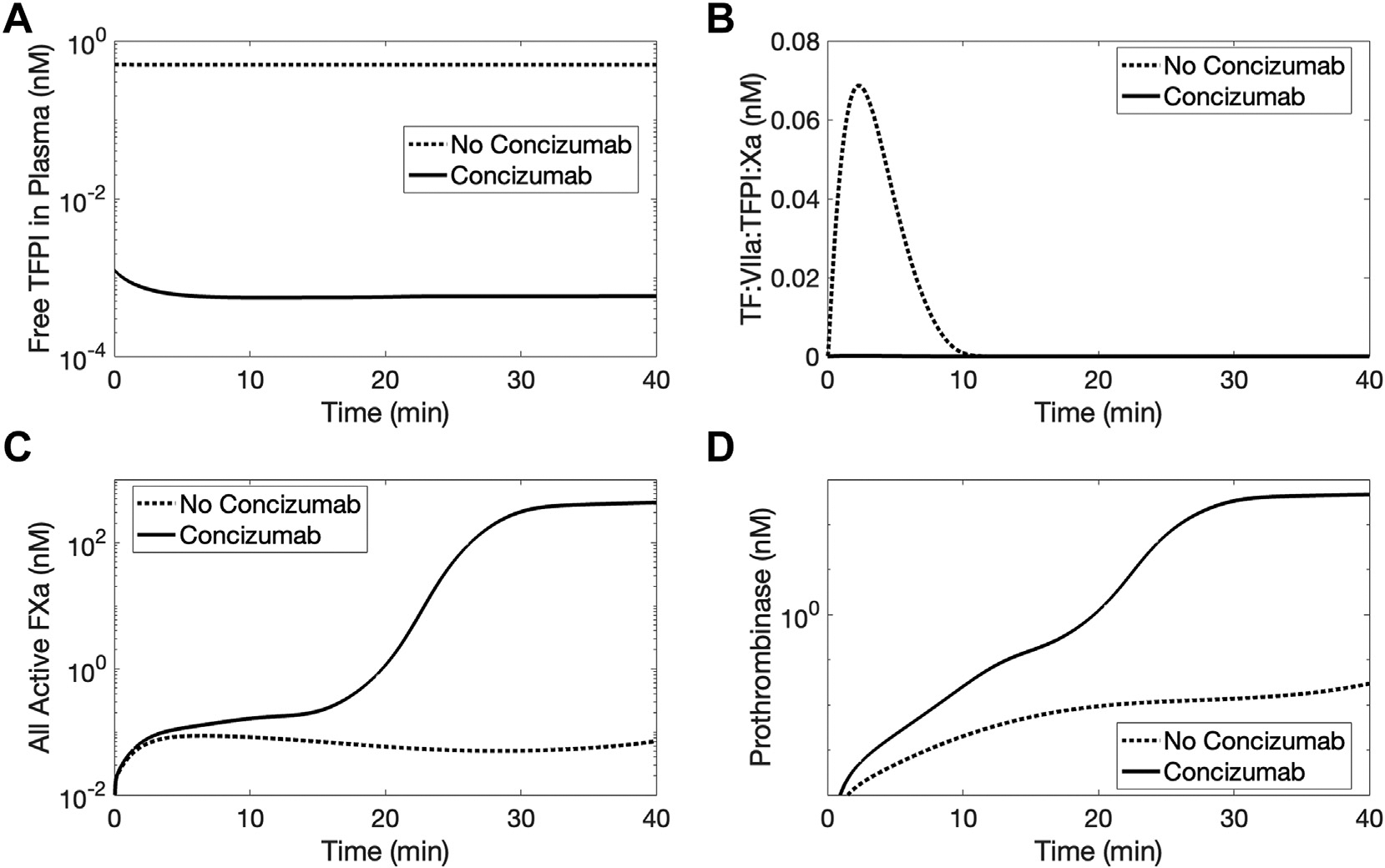
Concentration time courses of selected model species during simulated, flow-mediated coagulation. The panels show free tissue factor (TF) pathway inhibitor (TFPI)α in plasma (A), the subendothelial TF:factor (F)VIIa:FXa:TFPI complex (B), active FXa both in plasma and bound to a platelet surface (C), and platelet-bound prothrombinase (FXa:FVa and FXa:FV-h; D). The total intravascular concizumab level was 21.5 nM (4.14 nM free plasma concizumab and complexes with TFPIα and TFPIβ). TF was fixed at 9 fmol/cm^2^, and FVIII was 1% (0.01 nM). Concizumab blocked the inhibitory action of TFPIα (A, B), which led to increases in FXa and prothrombinase (C, D).

Our mathematical models actively tracked and observed all coagulation proteins, inhibitors, and complexes as they interacted and evolved through time. This modeling is complementary to wet-lab experimental approaches where typically only 1 output, usually thrombin, can be measured at a time. We simulated thrombin generation under hemophilia A conditions for 40 minutes using the intermediate TF level (9 fmol/cm^2^). The effects of concizumab on coagulation biochemistry were probed by viewing changes in 4 important proteins/complexes over time: free plasma TFPIα, TF:FVIIa:FXa:TFPIα, total FXa (free and bound to platelet surfaces), and prothrombinase (FXa:FVa and FXa:FV-h; Figure 4). concizumab reduced the amount of free TFPIα in plasma more than 100-fold (Figure 4A) and hindered the formation of TF:FVIIa:FXa:TFPI quaternary complex during the first 10 minutes of the simulation (Figure 4B). In the model, we assumed that platelets could adhere to the subendothelial surface exposed upon injury but not the intact endothelial surface. This adhesion effectively “covered” the subendothelium, reducing biochemical activity there. This reduced the formation of TF:FVIIa:FXa:TFPI after 10 minutes. Platelet surfaces then became the major source of FXa production in a process that was enhanced by concizumab. Concizumab enhanced FXa production by altering coagulation biochemistry in 2 steps. First, it dampened the inhibition of TF:FVIIa:FXa by TFPI on the subendothelium (Figure 4B), which increased the production of FVIIIa:FIXa (Supplementary Figure S2). With 1% FVIII, there was substantially less FVIIIa:FIXa than with 100% FVIII, but there were still small amounts present that increased 5 orders of magnitude with concizumab (Supplementary Figure S2). Consequently, more platelet-bound FXa (FXa^m^) was produced. Second, this FXa pool was protected from TFPI inhibition by C, with a total increase in FXa of 100-fold (Supplementary Figure S2). At 20 minutes, concizumab boosted total FXa generation by more than 1000-fold (Figure 4C). The FXa then assembled into prothrombinase complexes (FXa:FVa and FXa:FV-h) on activated platelet surfaces, resulting in a 10 000-fold increase in prothrombinase production compared with scenarios without C (Figure 4D).

### 3.4 | Concizumab promotes thrombin generation by blocking TFPI inhibitory activity at several locations

**FIGURE 5:**
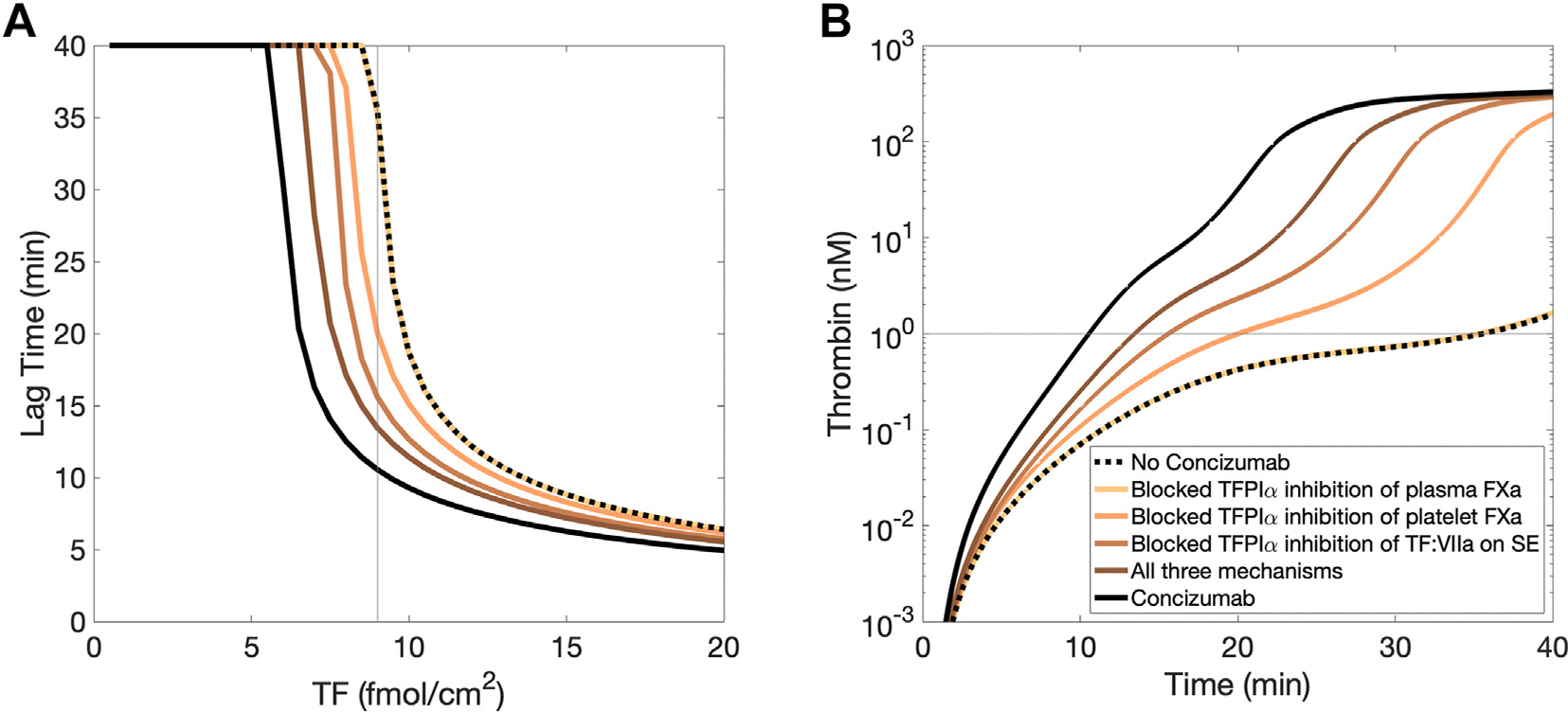
Thrombin generation as a function of tissue factor (TF) concentration and concizumab’s TF pathway inhibitor (TFPI) inhibitory mechanisms. Lag time (time to 1 nM thrombin) as a function of TF (A) and thrombin time courses (B) for various downstream mechanisms of concizumab at a fixed TF level of 9 fmol/cm^2^. Factor (F)VIII is fixed at 1%, and no other factors/inhibitor levels were varied. A thin gray line represents TF = 9 fmol/cm^2^ in (A) and 1 nM thrombin in (B). SE, subendothelium. Note that the points of intersection are the same data in both plots.

The TFPI K2 domain tightly binds FXa in plasma, on activated platelet surfaces, and at the subendothelium. Thus, at each of these locations, it can dampen thrombin generation by directly inhibiting FXa activity, providing feedback inhibition of TF:FVIIa, and inhibiting prothrombinase assembled with FV-h. The relative effect of TFPI inhibitory activity at individual locations on overall thrombin generation was examined in the mathematical model by toggling on or off selected inhibitory reactions or mechanisms. The time required to generate 1 nM thrombin (lag time) at low FVIII (1%) was assessed as a function of TF for 6 different scenarios: (1) no concizumab, all other reactions intact; (2) no concizumab, turning off TFPIα binding to plasma FXa; (3) no concizumab, turning off TFPIα binding to FXa^m^ and to FXa within FXa:FV-h on the platelet surface; (4) no concizumab, turning off TFPIα binding TF:FVIIa:FXa, and TFPI:FXa binding to TF:FVIIa at the subendothelium; (5) no C, combining scenarios 2 to 4; and (6) with concizumab and all other reactions intact. The lag time was decreased by concizumab at all TF concentrations between 1 and 20 fmol/cm^2^ (Figure 5A). Blocking TFPIα binding to plasma FXa had a negligible effect, as plasma FXa is quickly carried away by flow. In contrast, blocking TFPIα inhibition of FXa on the platelet surface and TF:FVIIa:FXa on the subendothelium had notable individual effects and subadditive effects when combined. At TF = 9 fmol/cm^2^, blocking TFPIα inhibition of FXa reduced the lag time to 20.1 minutes (~44% decrease), while blocking TFPIα inhibition of TF:FVIIa:FXa reduced it to 15.6 minutes (~56% decrease). Combined, these mechanisms shortened the lag time to 13.5 minutes (~62% decrease), though still longer than with concizumab (10.6 minutes, ~70% decrease). The difference arises from the residual inhibitory effect of TFPIα on FV-h at 0.5 nM. With concizumab, free TFPIα and C:TFPIα was only 0.09 nM (20% of 0.4328 nM, see Supplementary Table S1), reducing the inhibitory effects on FV-h, as shown in the next section.

Time courses for thrombin generation mimicked the trends observed for lag times using the scenario with TF at 9 fmol/cm^2^ (Figure 5B) but also revealed that a similar maximal thrombin concentration (200–300 nM) was present regardless of the TFPI inhibition mechanism that was blocked. Thus, these data indicate that concizumab promotes the rate of thrombin generation by inhibiting TFPI in plasma, on platelets, and on the subendothelium, with no location having a dominant effect.

### 3.5 | Concizumab, TFPIβ, and FV-h

**FIGURE 6:**
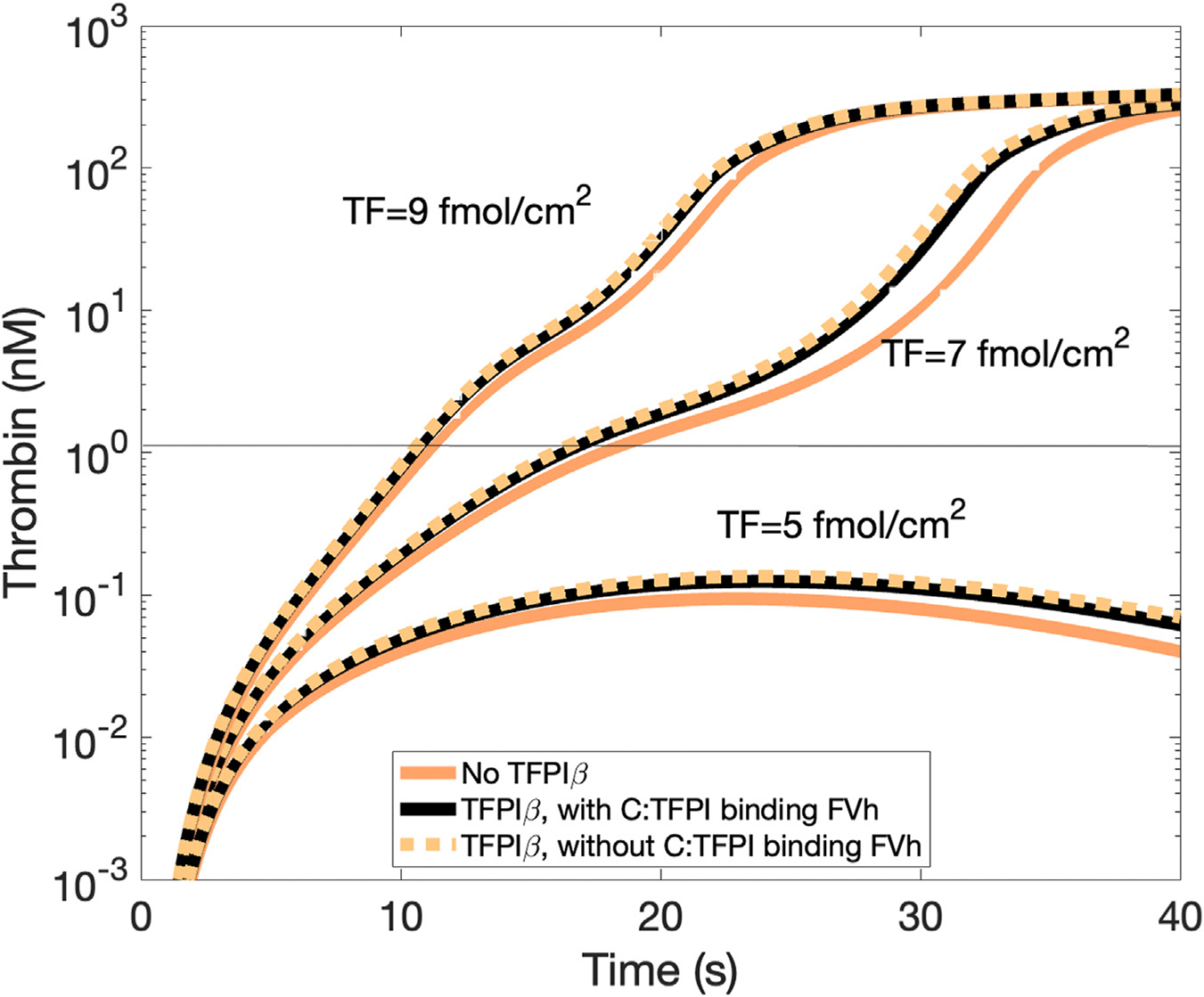
Thrombin generation time courses with concizumab (C), tissue factor (TF) pathway inhibitor (TFPI)β, and varied factor (F)V-“half” (FVh) interactions. The solid black curve for TF = 9 fmol/cm^2^ in this figure and Figure 5B is the same and shows simulations with both concizumab and TFPIβ. The orange solid curves are the same scenario but in the absence of TFPIβ. The dashed lines are with TFPIβ, but the binding of C:TFPIα to FVh was disabled. FVIII was set to 1% (0.01 nM).

TFPIα binds to FV-h with its C-terminus and to concizumab via the K2 domain. Thus, it is plausible that C:TFPIα could bind to FV-h. This means that C:TFPIα could retain some anticoagulant function by slowing the interaction of FXa and FV-h during prothrombinase assembly. Therefore, we examined the inhibitory nature of plasma C:TFPIα and compared it with that of TFPIβ:C:TFPIα. In these studies, thrombin generation was simulated under flow with TFPIβ set to 0 nM (total intravascular concizumab 4 nM) and TFPIβ set to 18 nM (total intravascular concizumab 21.5 nM), both with or without C:TFPIα binding to FV-h. Under these conditions, plasma C:TFPIα was 0.09 nM (with TFPIβ) and 0.26 nM (without TFPIβ). When TF was at 5, 7, and 9 fmol/cm^2^, the thrombin lag times were slightly shorter without TFPIβ than with TFPIβ. For example, at TF = 7 fmol/cm^2^, the lag time was 16.5 minutes without TFPIβ and 18 minutes with it. Similarly, at TF = 9 fmol/cm^2^, the lag time was 10 minutes without TFPIβ and 11 minutes with it. At TF = 5 fmol/cm^2^, thrombin levels remained below 1 nM (Figure 6). These delays were due solely to the inhibition of FV-h by plasma C:TFPIα. In the presence of TFPIβ, the inhibition of FV-h was negligible because there was only 0.09 nM C:TFPIα. These findings indicate that C:TFPIα binding to FV-h inhibited thrombin generation, although its role was minor.

### 3.6 | Concizumab procoagulant activity was not altered by protein S cofactor activity toward TFPIα

**FIGURE 7:**
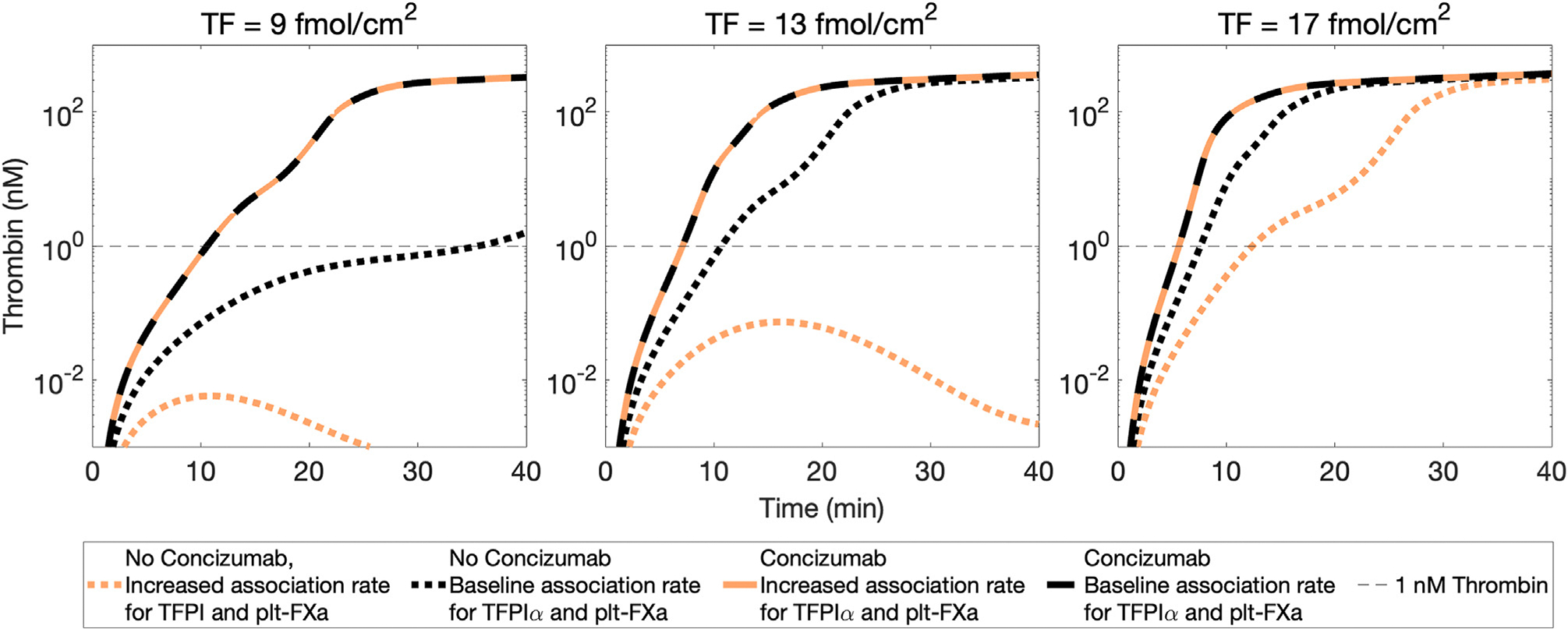
The simulated effects of protein S on thrombin generation. Thrombin time courses for tissue factor (TF) at 9, 13, and 17 fmol/cm^2^ with 1% factor (F)VIII (0.01 nM). Black curves are without protein S (baseline association rate between TF pathway inhibitor [TFPI]α and platelet-bound FXa [plt-FXa]). Orange curves are with protein S (increased associate rate). The dotted black and orange curves are without concizumab. Solid curves (with concizumab) are on top of each other, so they appear dashed.

Protein S acts as a cofactor for TFPIα [27,28], enhancing the inhibition of FXa and potentially influencing the procoagulant activity of concizumab. Protein S binds lipid surfaces and localizes TFPIα there, making it a more efficient inhibitor of FXa^m^ [29]. To recapitulate and test the effects of protein S on concizumab-mediated thrombin generation in hemophilia A, the flow-mediated model was modified by scaling kinetic rates to reflect thrombin generation in the presence or absence of protein S to examine its effects in hemophilia A with concizumab. We systematically increased the baseline association rate between TFPIα and FXa^m^, or soon-to-be FXa^m^, by factors of 10 until thrombin generation was abrogated for TF = 9 fmol/cm^2^. This was done by increasing the association rate in 4 reactions: TFPIα binding to FXa^m^, TFPIα:FV-h binding to FXa^m^, TFPIα binding to the FXa^m^ within the FXa:FV-h complex, and plasma FXa binding to platelet-bound TFPIα:FV-h. Together, these adjustments account for protein S cofactor activity toward TFPIα [27,28], as well as the formation of the trimolecular complex of protein S, TFPIα, and FV-h [18,30–32]. Scaling this association rate by a factor of 10 nearly eliminated thrombin generation in the absence of concizumab at TF = 9 fmol/cm^2^ (Figure 7A). Further, substantial thrombin was not reached with TF = 13 fmol/cm^2^ (Figure 7B); TF needed to be increased to 17 fmol/cm^2^ to obtain 1 nM thrombin by 40 minutes, and even then, the lag time was lengthened from 7.5 minutes to 12.5 minutes (Figure 7C). In the presence of concizumab, however, scaling the association rate to simulate the effects of protein S did not alter thrombin generation over all TF levels simulated. These results suggest that the procoagulant activity of concizumab is not altered by protein S cofactor activity toward TFPIα.

### 3.7 | Variation in affinity constants for concizumab binding to TFPIα and TFPIβ did not substantially alter thrombin generation

Different dissociation rates (K_D_) for concizumab binding to TFPIα and TFPIβ have been reported, likely due to variations in assay conditions. These include measured K_D_ values of 25 ± 8 pM for full-length TFPIα, 73 ± 13 pM for the isolated K2 domain, 123 pM [11] for TFPIβ using ^125^I-labeled concizumab, and 420 pM for TFPIβ using competitive enzyme-linked immunosorbent assay [12,33]. Fluctuations in the K_D_ may affect the steady-state concentration estimates from the compartment model, which in turn could affect thrombin generation under flow. The K_D_ was varied for concizumab binding to TFPIα from 15 to 100 pM and for concizumab binding to TFPIβ from 100 to 500 pM to examine the effects of uncertainties in the true K_D_ value.

The total plasma concizumab, free plasma TFPIα, and TFPIα sequestered to the endothelium through concizumab and TFPIβ for the specified K_D_ ranges were computed (Supplementary Figure S2A–C). Then, these steady-state concentrations were used in the flow model to compute the corresponding lag times (Supplementary Figure S2D). K_D_ variations had minimal effects on steady-state concentrations in the compartment model (total plasma concizumab range, 4–5 nM; free plasma TFPIα range, 0.002–0.0055 nM; TFPIα:C:TFPIβ range, 1.80–1.96 nM). The resulting variation of lag times in the thrombin generation model was also minimal (varied within a range of 10.55–10.65 minutes). Thus, variation of the K_D_ within previously published ranges has little effect on simulated thrombin generation.

## 4 | DISCUSSION

As new nonreplacement therapies emerge in the field of hemophilia treatment, there is a growing need to understand their underlying mechanisms of action. Concizumab is one of these nonfactor products targeting TFPI, the principal inhibitor of the early phases of coagulation. In the present study, mathematical modeling was used to simulate mechanisms of concizumab action on TFPI involved in multiple enzymes, enzyme complexes, and biological surfaces. The *in silico* thrombin generation flow model [21,22] is uniquely designed to do this, as all possible downstream effects of preventing TFPI inhibition of FXa can be addressed simultaneously. Furthermore, the model allowed platelets to cover the subendothelium and reduce enzymatic activity at the injury site, reflecting the dynamic processes initiated by a small vessel injury. Therefore, competition is present between the generation of FIXa and FXa by TF:FVIIa and the platelets covering TF:FVIIa at the subendothelium. The model also enabled the assembly of FVIIIa:FIXa on platelets, though this assembly was less efficient with low FVIII levels compared with normal levels. This tenase complex acts as an additional mechanism to generate FXa and support more robust thrombin generation. When the TF concentration was low, platelets won the competition and covered the “injury site,” resulting in a very slow increase of thrombin that did not reach 1 nM thrombin, the concentration that activates platelets. At higher TF concentrations, thrombin reached 1 nM and then continued to increase as platelets were activated and provided active surfaces where coagulation occurred. Since the model did not consider fibrin dynamics or fibrinolysis, there was a constant supply of flowing substrates, and the thrombin curves increased over time at a steady rate.

Concizumab enhances thrombin generation through tight binding to the TFPI K2 domain in a manner that blocks TFPI-K2 from binding to FXa. Since TFPI is a FXa-dependent inhibitor of TF:FVIIa, concizumab also blocks efficient inhibition of TF:FVIIa by TFPI, which is a primary mechanism of concizumab activity [12]. Consistent with this mechanism of action, concizumab enhanced thrombin generation across a range of TF concentrations. However, its procoagulant potential varied depending on the amount of TF present. At low TF (<6 fmol/cm^2^), concizumab increased thrombin generation, but it did not exceed 1 nM after 40 minutes. At intermediate TF (6–10 fmol/cm^2^), concizumab substantially increased the thrombin produced (>1 nM after 40 minutes) and shortened lag times. At higher TF (>10 fmol/cm^2^), concizumab also reduced the lag times, but final thrombin concentrations were not further increased. This was due to the compensatory effect of high TF concentrations, which offset FVIII deficiency by promoting thrombin generation solely through the extrinsic pathway. These results are in line with our previous simulations, where TFPI delays the initiation of thrombin generation in plasma with normal FVIII concentration but has minimal impact on final thrombin concentrations [21]. The results from the present study demonstrated that with FVIII deficiency, concizumab had the largest relative reduction in lag times at the intermediate TF concentrations.

TFPI has other important physiological anticoagulant activities than inhibition of TF:FVIIa:FXa, including direct inhibition of FXa on the platelet surface by TFPIα [21] and the inhibition of early forms of prothrombinase. Here, we used mathematical models to probe 4 mechanisms through which concizumab-mediated inhibition of TFPI anticoagulant activity could enhance thrombin generation: 1) blocking inhibition of FXa in the plasma; 2) blocking inhibition of TF:FVIIa at the subendothelium; 3) blocking inhibition of FXa and FXa:FVh at the platelet surface; and 4) sequestration of plasma TFPIα to the endothelium through formation of the ternary TFPIα:C:TFPIβ complex. Except for the inhibition of plasma FXa alone, the flow model predicted that each of the other individual mechanisms would reduce the lag time for thrombin generation in TF-initiated reactions. Thus, our data indicate that the procoagulant activity of concizumab is mediated by events occurring at 3 cellular surfaces: 1) the site of TF exposure on the injured subendothelium, 2) the surface of activated platelets, and 3) the intact endothelium expressing TFPIβ. Of these, the sequestration of plasma TFPIα to the endothelium had the most significant impact, accounting for 75% of concizumab procoagulant activity.

TFPIβ on the endothelium bound large amounts of concizumab, either free concizumab or the C:TFPIα complex, which occurred because we considered the possibility that concizumab could bind TFPIα with one arm and TFPIβ with its other arm. This possibility is supported by the dual concizumab binding sites for the K2 domain [12,20] and the flexible nature of TFPIα [34]. Allowing binding to TFPIβ greatly reduced the amount of C:TFPIα circulating in the plasma. Thus, the model predicted that TFPIβ had an overall procoagulant effect in the presence of concizumab by removing TFPIα from the plasma. This finding is difficult or impossible to test using *ex vivo* biochemical studies but can easily be examined in our mathematical models, providing an example of their utility.

Since TFPIα inhibits early forms of prothrombinase containing FV-h, we investigated how concizumab may alter TFPIα binding to FV-h. Previous work indicated that TFPIα lacking FXa inhibitory capacity exerts anticoagulant activity by slowing prothrombinase assembly [35]. Our mathematical simulations showed that the TFPIα:C complex slowed thrombin generation, which is consistent with the basic C-terminus of TFPIα retaining its ability to interact with FV-h when concizumab is bound to the K2 domain. However, this was a relatively minor effect compared with other concizumab mechanisms of action studied because of the small amount of TFPIα:C complex remaining in plasma.

As with any model—whether *in silico*, *in vitro*, or otherwise—there are limitations. This model, for instance, cannot account for injuries larger than ~10 μm or corresponding spatial variations, nor does it consider blood loss in the case of bleeding. It also excludes fibrinogen, the generation of fibrin by thrombin, and fibrin polymerization. Additionally, as previously shown [36], endothelial reactions are assumed to occur in an RZ adjacent to the subendothelial zone and are subjected to flow. Consequently, they have minimal impact on subendothelial reactions when exposed to flow. Thus, we assumed that the primary role of TFPIβ in the model was to affect the plasma concentrations of TFPIα by forming ternary complexes with it and concizumab in the steady-state model, and we did not include it as an explicit variable in the flow-mediated model. However, in cases of larger injuries or scenarios where the endothelial and subendothelial zones overlap, TFPIβ and endothelial reactions may play a more significant role. Future work could explore this by integrating concizumab-related reactions into our previously developed spatiotemporal models, which investigate the spatial effects of flow and larger patches of mixed subendothelium and endothelium. Nonetheless, the mathematical model used in this study offers valuable insight. It was built on a comprehensive list of coagulation reactions and activated platelet surfaces and has consistently predicted experimentally verified responses to hemostatic perturbations [26,37–40].

## 5 | CONCLUSION

Our mathematical simulations investigating the effects of concizumab on thrombin generation on multiple cellular surfaces comprised 2 submodels: a simple steady-state model for estimating drug plasma levels and a flow-mediated coagulation model for simulating how concizumab affects thrombin generation. Together, these models simulated how concizumab promotes hemostasis at locations close to the subendothelium where TF is exposed following vascular injury, such as by promoting FXa generation on the surface of activated platelets and by allowing TFPIβ to sequester C:TFPIα complexes out of plasma to the endothelial surface. The models found that concizumab enhanced the initiation and amplification of coagulation, thereby shortening thrombin generation lag times under flow. Reduction of plasma TFPIα concentration was the largest driver of concizumab procoagulant activity, while blocking TFPIα-mediated inhibition of TF:FVIIa and FXa^m^ also contributed to the reduction in lag times. These procoagulant effects were most evident at intermediate TF levels used in the model, where thrombin generation was sensitive to the TF concentration, and small enhancements in FXa shifted the system from the subnanomolar thrombin generation observed at the low TF concentrations to the robust thrombin burst observed at high TF concentration. Our description of the multiple biochemical mechanisms and sites of action through which concizumab promotes hemostasis further explains its efficacy for hemophilia prophylaxis.

## Supplementary Material

supplemental

The online version contains supplementary material available at https://doi.org/10.1016/j.jtha.2024.10.028

## Figures and Tables

**TABLE:** Biochemical reactions and corresponding parameter values for mathematical models.

Reaction list for compartment model						

#	Reactants	Products	Parameter(s)	*k*^+^ (*M*^−1^*s*^−1^)	*k*^−^ (s^−1^)	Note

C1	C + TFPIα	C:TFPIα	$k_{\alpha }^{+},\;k_{\alpha }^{-}$	4.48 × 10^6^	1 × 10^−4^	a
C2	C:TFPIα + TFPIα	TFPIα:C:TFPIα	$k_{\alpha }^{+},\;k_{\alpha }^{-}$	4.48 × 10^6^	1 × 10^−4^	a
C3	C + TFPIβ	C:TFPIβ	$k_{\beta }^{+},\;k_{\beta }^{-}$	8.21 × 10^5^	1 × 10^−4^	b
C4	C:TFPIα + TFPIβ	TFPIα:C:TFPIβ	$k_{\beta }^{+},\;k_{\beta }^{-}$	8.21 × 10^5^	1 × 10^−4^	b
C5	TFPIα + C:TFPIβ	TFPIα:C:TFPIβ	$k_{\alpha }^{+},\;k_{\alpha }^{-}$	4.48 × 10^6^	1 × 10^−4^	a

Reaction list for flow model						

#	Reactant	Product	Parameter(s)	(*M*^−1^*s*^−1^)	(*s*^−1^)	Note

1a	TF:FVIIa + FX	TF:FVIIa:FX	$k_{X}^{+},\;k_{X}^{-}$	4.35 × 10^6^	1	a
1b	TF:FVIIa:FX	TF:FVIIa:FXa	$k_{\mathrm{cat}}$		4.6	a
2	TF:FVIIa + FXa	TF:FVIIa:FXa	$k_{Xa}^{+},\;k_{Xa}^{-}$	1.92 × 10^6^	1	a
3	TF:FVIIa:FXa + TFPIα	TF:FVIIa:FXa:TFPIα	$k_{\alpha Xa}^{+},\;k_{\alpha Xa}^{-}$	1.6 × 10^7^	3.3 × 10^−4^	b
4	C + TFPIα	C:TFPIα	$k_{\alpha }^{+},\;k_{\alpha }^{-}$	4 × 10^6^	1 × 10^−4^	c
5	C:TFPIα + TFPIα	TFPIα:C:TFPIα	$k_{\alpha }^{+},\;k_{\alpha }^{-}$	4 × 10^6^	1 × 10^−4^	c
6	C + TFPIα:FV^h^	C:TFPIα:FV^h^	$k_{\alpha }^{+},\;k_{\alpha }^{-}$	4 × 10^6^	1 × 10^−4^	c
7	C:TFPIα + FV^h^	C:TFPIα:FV^h^	$k_{Vh}^{+},\;k_{Vh}^{-}$	5 × 10^7^	4.5 × 10^−3^	d
8	C + TFPIα:FV^hm^	C:TFPIα:FV^hm^	$k_{\alpha }^{+},\;k_{\alpha }^{-}$	4 × 10^6^	1 × 10^−4^	c
9	C:TFPIα + FV^hm^	C:TFPIα:FV^hm^	$k_{Vh}^{+},\;k_{Vh}^{-}$	5 × 10^7^	4.5 × 10^−3^	d
10	C + TFPIα:FV^hm^:FXa^m^	C:TFPIα:FV^hm^:FXa^m^	$k_{\alpha }^{+},\;k_{\alpha }^{-}$	4 × 10^6^	1 × 10^−4^	c

Top: list of reactions included in the compartment model. C represents concizumab. Notes: kinetic rates are taken from a) binding of concizumab to TFPIα, dissociation rate (K_D_) = 25 pM [12]. b) Binding of concizumab to TFPIβ, K_D_ = 123 pM [12]. Bottom: list of reactions included in the extended flow-mediated coagulation model. Superscript h indicates the partially cleaved form of FV (FV-h). Superscript m indicates that the model species is bound to a platelet membrane. Parameters separated by columns have values listed in the following 2 columns, respectively.

Notes:

a) activation of FX by TF:FVIIa from Lu et al. [41]

b) TFPIα binding FXa, K_D_ = 20 pM from Jesty et al. [19];

c) concizumab binding TFPIα from Hilden et al. [12]

d) binding of TFPIα to FV-h, K_D_ = 9 pM from Wood et al. [16].

Reactions 4 and 5 in the flow model (bottom) are the same as reactions C1 and C2 in the compartment model (top). FV/FVIIa/FXa, factor V/activated factor VII/factor Xa; TF, tissue factor; TFPI, tissue factor pathway inhibitor.
