# Supplementary material for: Examining downstream effects of concizumab in hemophilia A with a mathematical modeling approach
Source: J Thromb Haemost. Author manuscript; Available in PMC 2025 Aug 11. (PMC12336664; doi:10.1016/j.jtha.2024.10.028)
Supplement: supplemental [file NIHMS2100267-supplement-supplemental.pdf]

Supplementary Information for: Examining Downstream Effects of Concizumab in Hemophilia A with a Mathematical Modeling Approach, Miyazawa et al.

1 Estimate of size of endothelial pool of TFPIβ

The total plasma TFPIα is 1.6 – 2.5 nM.[1] Heparin administration increases plasma TFPIα concentration 1.5 to 4-fold. i.e. to 2-10 nM due to release of glycosaminoglycan-bound TFPIα from the endothelium including the extracellular matrix.[2] In cell experiments, phosphatidylinositol phospholipase C, which cleaves off GPI-anchored proteins from the cell, releases 80% of cell surface TFPI corresponding to TFPIβ.[3] The remaining TFPIα can be released by heparin. This indicates a 20:80 distribution between endothelial TFPIα and TFPIβ, and that the total TFPI (α and β) in the endothelium is five-fold that of releasable TFPIα. Assuming that the cell experiments parallel in vivo conditions, the total endothelial pool of TFPI would be five-fold higher than the heparin releasable TFPIα pool, i.e. 10-50 nM. TFPIβ corresponds to 80% of this, i,e., to 8-40 nM (average value 24 nM).

In a systems pharmacokinetic/pharmacodynamic model based on non-clinical and clinical phase 1 data for concizumab,[4] the membrane-bound (endothelial) pool of TFPI was estimated to be 20 nM with a coefficient of variation of 13%. Assuming that the membrane pool of TFPI also comprises extracellular matrix TFPI, TFPIβ would comprise 80% of the total, membrane bound TFPI, i.e. 16 nM.

In conclusion, the TFPIβ concentration is estimated to be somewhere between 8 and 40 nM, with modelling data pointing to 16 nM. In the present study, a TFPIβ concentration of 18 nM was used to account for both the model estimate and the estimate based on heparin-releasable TFPI.

2 Compartment Model Diagram, Reactions & Equations

2.1 Network Diagram

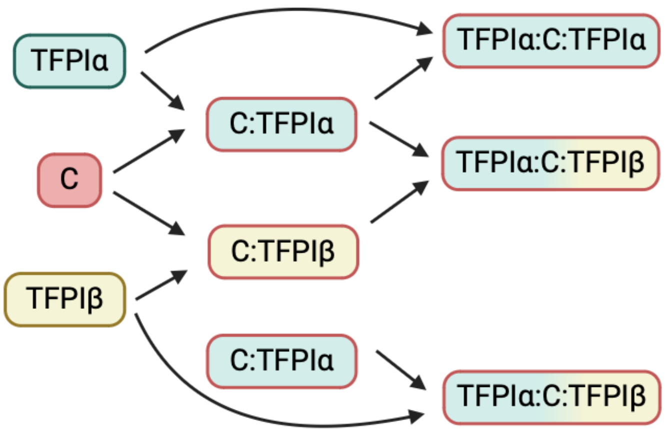

Figure S1: Schematic of reactions in compartment model. Blue represents TFPIα, red or red border represents concizumab , and yellow represents TFPIβ.

2.2 Reactions

$$\mathrm{C} + \mathrm{TFPI}\alpha \underset{k_\alpha^-}{\overset{k_\alpha^+}{\longleftrightarrow}} \mathrm{C{:}TFPI}\alpha \tag{1}$$

$$\mathrm{C{:}TFPI}\alpha + \mathrm{TFPI}\alpha \underset{k_\alpha^-}{\overset{k_\alpha^+}{\longleftrightarrow}} \mathrm{TFPI}\alpha\mathrm{{:}C{:}TFPI}\alpha \tag{2}$$

$$\mathrm{C} + \mathrm{TFPI}\beta \underset{k_\beta^-}{\overset{k_\beta^+}{\longleftrightarrow}} \mathrm{C{:}TFPI}\beta \tag{3}$$

$$\mathrm{C{:}TFPI}\alpha + \mathrm{TFPI}\beta \underset{k_\beta^-}{\overset{k_\beta^+}{\longleftrightarrow}} \mathrm{TFPI}\alpha\mathrm{{:}C{:}TFPI}\beta \tag{4}$$

$$\mathrm{TFPI}\alpha + \mathrm{C{:}TFPI}\beta \underset{k_\alpha^-}{\overset{k_\alpha^+}{\longleftrightarrow}} \mathrm{TFPI}\alpha\mathrm{{:}C{:}TFPI}\beta \tag{5}$$

2.3 Ordinary Differential Equations

$$\frac{d[\mathrm{C}]}{dt} = -k_\alpha^+[\mathrm{C}][\mathrm{TFPI}\alpha] + k_\alpha^-[\mathrm{C{:}TFPI}\alpha] - k_\beta^+[\mathrm{C}][\mathrm{TFPI}\beta] + k_\beta^-[\mathrm{C{:}TFPI}\beta] \tag{6}$$

$$\begin{aligned}\frac{d[\mathrm{TFPI}\alpha]}{dt} &= -k_\alpha^+[\mathrm{C}][\mathrm{TFPI}\alpha] + k_\alpha^-[\mathrm{C{:}TFPI}\alpha] - k_\alpha^+[\mathrm{C{:}TFPI}\alpha][\mathrm{TFPI}\alpha] + k_\alpha^-[\mathrm{TFPI}\alpha\mathrm{{:}C{:}TFPI}\alpha] \\ &\quad - k_\alpha^+[\mathrm{TFPI}\alpha][\mathrm{TFPI}\beta] + k_\alpha^-[\mathrm{TFPI}\alpha\mathrm{{:}C{:}TFPI}\beta]\end{aligned} \tag{7}$$

$$\begin{aligned}\frac{d[\mathrm{C{:}TFPI}\alpha]}{dt} &= k_\alpha^+[\mathrm{C}][\mathrm{TFPI}\alpha] - k_\alpha^-[\mathrm{C{:}TFPI}\alpha] - k_\alpha^+[\mathrm{C{:}TFPI}\alpha][\mathrm{TFPI}\alpha] + k_\alpha^-[\mathrm{TFPI}\alpha\mathrm{{:}C{:}TFPI}\alpha] \\ &\quad - k_\beta^+[\mathrm{C{:}TFPI}\alpha][\mathrm{TFPI}\beta] + k_\beta^-[\mathrm{TFPI}\alpha\mathrm{{:}C{:}TFPI}\beta]\end{aligned} \tag{8}$$

$$\frac{d[\mathrm{TFPI}\alpha\mathrm{{:}C{:}TFPI}\alpha]}{dt} = k_\alpha^+[\mathrm{C{:}TFPI}\alpha][\mathrm{TFPI}\alpha] - k_\alpha^-[\mathrm{TFPI}\alpha\mathrm{{:}C{:}TFPI}\alpha] \tag{9}$$

$$\frac{d[\mathrm{TFPI}\beta]}{dt} = -k_\beta^+[\mathrm{C}][\mathrm{TFPI}\beta] + k_\beta^-[\mathrm{C{:}TFPI}\beta] - k_\beta^+[\mathrm{C{:}TFPI}\alpha][\mathrm{TFPI}\beta] + k_\beta^-[\mathrm{TFPI}\alpha\mathrm{{:}C{:}TFPI}\beta] \tag{10}$$

$$\frac{d[\mathrm{C{:}TFPI}\beta]}{dt} = k_\beta^+[\mathrm{C}][\mathrm{TFPI}\beta] - k_\beta^-[\mathrm{C{:}TFPI}\beta] - k_\alpha^+[\mathrm{TFPI}\alpha][\mathrm{C{:}TFPI}\beta] + k_\alpha^-[\mathrm{TFPI}\alpha\mathrm{{:}C{:}TFPI}\beta] \tag{11}$$

$$\begin{aligned}\frac{d[\mathrm{TFPI}\alpha\mathrm{{:}C{:}TFPI}\beta]}{dt} &= k_\beta^+[\mathrm{C{:}TFPI}\alpha][\mathrm{TFPI}\beta] - k_\beta^-[\mathrm{TFPI}\alpha\mathrm{{:}C{:}TFPI}\beta] \\ &\quad + k_\alpha^+[\mathrm{TFPI}\alpha][\mathrm{C{:}TFPI}\beta] - k_\alpha^-[\mathrm{TFPI}\alpha\mathrm{{:}C{:}TFPI}\beta]\end{aligned} \tag{12}$$

2.4 Python code to simulate compartment model

```python
import numpy as np
from scipy.integrate import odeint
import matplotlib.pyplot as plt

# Define the system of ODEs
def compartment_model(y, t, k1, k2, k3, k4):
    C, TFPIa, C_TFPIa, TFPIa_C_TFPIa, TFPIB, C_TFPIB, TFPIa_C_TFPIB = y

    dC_dt = -k1 * C * TFPIa + k2 * C_TFPIa - k3 * C * TFPIB + k4 * C_TFPIB
    dTFPIa_dt = -k1 * C * TFPIa + k2 * C_TFPIa - k1 * C_TFPIa * TFPIa \
      + k2 * TFPIa_C_TFPIa - k1 * TFPIa * C_TFPIB + k2 * TFPIa_C_TFPIB
    dC_TFPIa_dt = k1 * C * TFPIa - k2 * C_TFPIa - k1 * C_TFPIa * TFPIa \
      + k2 * TFPIa_C_TFPIa - k3 * C_TFPIa * TFPIB + k4 * TFPIa_C_TFPIB
    dTFPIa_C_TFPIa_dt = k1 * C_TFPIa * TFPIa - k2 * TFPIa_C_TFPIa
```

```python
    dTFPIB_dt = -k3 * C * TFPIB + k4 * C_TFPIB - k3 * C_TFPIa * TFPIB \
      + k4 * TFPIa_C_TFPIB
    dC_TFPIB_dt = k3 * C * TFPIB - k4 * C_TFPIB - k1 * TFPIa * C_TFPIB \
      + k2 * TFPIa_C_TFPIB
    dTFPIa_C_TFPIB_dt = k3 * C_TFPIa * TFPIB - k4 * TFPIa_C_TFPIB \
      + k1 * TFPIa * C_TFPIB - k2 * TFPIa_C_TFPIB

    return [dC_dt, dTFPIa_dt, dC_TFPIa_dt, dTFPIa_C_TFPIa_dt, dTFPIB_dt,\
            dC_TFPIB_dt, dTFPIa_C_TFPIB_dt]

# Initial conditions
C0 = 21.5
TFPIa0 = 2.5
C_TFPIa0 = 0.0
TFPIa_C_TFPIa0 = 0.0
TFPIB0 = 18.0
C_TFPIB0 = 0.0
TFPIa_C_TFPIB0 = 0.0

y0 = [C0, TFPIa0, C_TFPIa0, TFPIa_C_TFPIa0, TFPIB0, C_TFPIB0, TFPIa_C_TFPIB0]

# Time points where solution is computed
t = np.linspace(0, 1000, 100)

# Parameters
k1 = 4.48e-3
k2 = 1.0e-4
k3 = 8.21e-4
k4 = 1.0e-4


# Solve ODEs
solution = odeint(compartment_model, y0, t, args=(k1, k2, k3, k4))

# Plot results
plt.plot(t, solution[:, 0], label='[C]')
plt.plot(t, solution[:, 1], label='[TFPIa]')
plt.plot(t, solution[:, 2], label='[C:TFPIa]')
plt.plot(t, solution[:, 3], label='[TFPIa:C:TFPIa]')
plt.plot(t, solution[:, 4], label='[TFPIB]')
plt.plot(t, solution[:, 5], label='[C:TFPIB]')
plt.plot(t, solution[:, 6], label='[TFPIa:C:TFPIB]')

plt.xlabel('Time (sec)')
plt.ylabel('Concentration (nM)')
plt.ylim(0, 26)
plt.legend(loc='upper right')
plt.show()
```

Species	Steady-state concentration in presence of TFPIβ (nM)	Steady-state concentration in absence of TFPIβ (nM)
Intravascular C	21.5	4.00
Total plasma C	4.14	4.00
C (free plasma)	3.59	2.09
TFPIα	2.80×10^{-3}	0.01
C:TFPIα	0.43	1.33
TFPIα:C:TFPIα	0.11	0.58
TFPIβ	0.63	0.00
C:TFPIβ	15.52	0.00
TFPIα:C:TFPIβ	1.85	0.00

Table S1: Steady-state concentrations of all compartment model species. Columns show results from total intravascular concizumab concentrations 21.5 nM (with TFPIβ) and 4 nM (without TFPIβ). These values correspond to the points highlighted in Figure 2A with an orange dot and a blue triangle. Note that for the TFPIα:C:TFPIα complex, the reported concentration is for the complex only; to recover the 2.5 nM total TFPIα, the concentration of the complex must be multiplied by two to account for both of the TFPIα molecules within the complex.

3 Flow Model Extension: Reactions & Equations

The model detailed in this study is an extension to our previous work [5, 6, 7, 8, 9]. Simulations of this model (in the absence of heparin and concizumab) can be performed with our online coagulation simulator: ClotSims. Below, we list the new reactions (from Table 2), their kinetic parameters, and the corresponding ordinary differential equations.

3.1 Reactions (17-23 are new to the flow model)

$$\text{TF:FVIIa} + \text{FX} \underset{k_X^-}{\overset{k_X^+}{\longleftrightarrow}} \text{TF:FVIIa:FX} \tag{13}$$

$$\text{TF:FVIIa:FX} \xrightarrow{k_{cat}} \text{TF:FVIIa:FXa} \tag{14}$$

$$\text{TF:FVIIa} + \text{FXa} \underset{k_{Xa}^-}{\overset{k_{Xa}^+}{\longleftrightarrow}} \text{TF:FVIIa:FXa} \tag{15}$$

$$\text{TF:FVIIa:FXa} + \text{TFPI}\alpha \underset{k_{\alpha Xa}^-}{\overset{k_{\alpha Xa}^+}{\longleftrightarrow}} \text{TF:FVIIa:FXa:TFPI}\alpha \tag{16}$$

$$\text{C} + \text{TFPI}\alpha \underset{k_\alpha^-}{\overset{k_\alpha^+}{\longleftrightarrow}} \text{C:TFPI}\alpha \tag{17}$$

$$\text{C:TFPI}\alpha + \text{TFPI}\alpha \underset{k_\alpha^-}{\overset{k_\alpha^+}{\longleftrightarrow}} \text{TFPI}\alpha\text{:C:TFPI}\alpha \tag{18}$$

$$\text{C} + \text{TFPI}\alpha\text{:FV}^h \underset{k_\alpha^-}{\overset{k_\alpha^+}{\longleftrightarrow}} \text{C:TFPI}\alpha\text{:FV}^h \tag{19}$$

$$\text{C:TFPI}\alpha + \text{FV}^h \underset{k_{Vh}^-}{\overset{k_{Vh}^+}{\longleftrightarrow}} \text{C:TFPI}\alpha\text{:FV}^h \tag{20}$$

$$\text{C} + \text{TFPI}\alpha\text{:FV}^{hm} \underset{k_\alpha^-}{\overset{k_\alpha^+}{\longleftrightarrow}} \text{C:TFPI}\alpha\text{:FV}^{hm} \tag{21}$$

$$\text{C:TFPI}\alpha + \text{FV}^{hm} \underset{k_{Vh}^-}{\overset{k_{Vh}^+}{\longleftrightarrow}} \text{C:TFPI}\alpha\text{:FV}^{hm} \tag{22}$$

$$\text{C} + \text{TFPI}\alpha\text{:FV}^{hm}\text{:FXa}^m \underset{k_\alpha^-}{\overset{k_\alpha^+}{\longleftrightarrow}} \text{C:TFPI}\alpha\text{:FV}^{hm}\text{:FXa}^m \tag{23}$$

3.2 Ordinary Differential Equations

$$\frac{d[\text{TF:FVIIa}]}{dt} = -k_X^+[\text{TF:FVIIa}][\text{FX}] + k_X^-[\text{TF:FVIIa:FX}] - k_{Xa}^+[\text{TF:FVIIa}][\text{FXa}] + k_{Xa}^-[\text{TF:FVIIa:FXa}] \tag{24}$$

$$\frac{d[\text{FX}]}{dt} = -k_X^+[\text{TF:FVIIa}][\text{FX}] + k_X^-[\text{TF:FVIIa:FX}] \tag{25}$$

$$\frac{d[\text{TF:FVIIa:FX}]}{dt} = k_X^+[\text{TF:FVIIa}][\text{FX}] - k_X^-[\text{TF:FVIIa:FX}] - k_{cat}[\text{TF:FVIIa:FX}] \tag{26}$$

$$\frac{d[\text{TF:FVIIa:FXa}]}{dt} = k_{cat}[\text{TF:FVIIa:FX}] + k_{Xa}^+[\text{TF:FVIIa}][\text{FXa}] - k_{Xa}^-[\text{TF:FVIIa:FXa}] - k_{\alpha Xa}^+[\text{TF:FVIIa:FXa}][\text{TFPI}\alpha] + k_{\alpha Xa}^-[\text{TF:FVIIa:FXa:TFPI}\alpha] \tag{27}$$

$$\frac{d[\text{FXa}]}{dt} = k_{cat}[\text{TF:FVIIa:FX}] + k_{Xa}^-[\text{TF:FVIIa:FXa}] - k_{Xa}^+[\text{TF:FVIIa}][\text{FXa}] \tag{28}$$

$$\frac{d[\text{TFPI}\alpha]}{dt} = -k_{\alpha Xa}^+[\text{TF:FVIIa:FXa}][\text{TFPI}\alpha] + k_{\alpha Xa}^-[\text{TF:FVIIa:FXa:TFPI}\alpha] - k_\alpha^+[\text{C}][\text{TFPI}\alpha] + k_\alpha^-[\text{C:TFPI}\alpha] - k_\alpha^+[\text{C:TFPI}\alpha][\text{TFPI}\alpha] + k_\alpha^-[\text{TFPI}\alpha\text{:C:TFPI}\alpha] \tag{29}$$

$$\frac{d[\text{TF:FVIIa:FXa:TFPI}\alpha]}{dt} = k_{\alpha Xa}^+[\text{TF:FVIIa:FXa}][\text{TFPI}\alpha] - k_{\alpha Xa}^-[\text{TF:FVIIa:FXa:TFPI}\alpha] \tag{30}$$

$$\frac{d[\text{C}]}{dt} = -k_\alpha^+[\text{C}][\text{TFPI}\alpha] + k_\alpha^-[\text{C:TFPI}\alpha] - k_\alpha^+[\text{C}][\text{TFPI}\alpha\text{:FV}^h] + k_\alpha^-[\text{C:TFPI}\alpha\text{:FV}^h] - k_\alpha^+[\text{C}][\text{TFPI}\alpha\text{:FV}^{hm}] + k_\alpha^-[\text{C:TFPI}\alpha\text{:FV}^{hm}] - k_\alpha^+[\text{C}][\text{TFPI}\alpha\text{:FV}^{hm}\text{:FXa}^m] + k_\alpha^-[\text{C:TFPI}\alpha\text{:FV}^{hm}\text{:FXa}^m] \tag{31}$$

$$\frac{d[\text{C:TFPI}\alpha]}{dt} = k_\alpha^+[\text{C}][\text{TFPI}\alpha] - k_\alpha^-[\text{C:TFPI}\alpha] - k_\alpha^+[\text{C:TFPI}\alpha][\text{TFPI}\alpha] + k_\alpha^-[\text{TFPI}\alpha\text{:C:TFPI}\alpha] \tag{32}$$

$$\frac{d[\text{TFPI}\alpha\text{:C:TFPI}\alpha]}{dt} = k_\alpha^+[\text{C:TFPI}\alpha][\text{TFPI}\alpha] - k_\alpha^-[\text{TFPI}\alpha\text{:C:TFPI}\alpha] \tag{33}$$

$$\frac{d[\text{TFPI}\alpha\text{:FV}^h]}{dt} = -k_\alpha^+[\text{C}][\text{TFPI}\alpha\text{:FV}^h] + k_\alpha^-[\text{C:TFPI}\alpha\text{:FV}^h] \tag{34}$$

$$\frac{d[\text{C:TFPI}\alpha\text{:FV}^h]}{dt} = k_\alpha^+[\text{C}][\text{TFPI}\alpha\text{:FV}^h] - k_\alpha^-[\text{C:TFPI}\alpha\text{:FV}^h] \tag{35}$$

$$\frac{d[\text{TFPI}\alpha\text{:FV}^{hm}]}{dt} = -k_\alpha^+[\text{C}][\text{TFPI}\alpha\text{:FV}^{hm}] + k_\alpha^-[\text{C:TFPI}\alpha\text{:FV}^{hm}] \tag{36}$$

$$\frac{d[\text{C:TFPI}\alpha\text{:FV}^{hm}]}{dt} = k_\alpha^+[\text{C}][\text{TFPI}\alpha\text{:FV}^{hm}] - k_\alpha^-[\text{C:TFPI}\alpha\text{:FV}^{hm}] \tag{37}$$

$$\frac{d[\text{TFPI}\alpha\text{:FV}^{hm}\text{:FXa}^m]}{dt} = -k_\alpha^+[\text{C}][\text{TFPI}\alpha\text{:FV}^{hm}\text{:FXa}^m] + k_\alpha^-[\text{C:TFPI}\alpha\text{:FV}^{hm}\text{:FXa}^m] \tag{38}$$

$$\frac{d[\text{C:TFPI}\alpha\text{:FV}^{hm}\text{:FXa}^m]}{dt} = k_\alpha^+[\text{C}][\text{TFPI}\alpha\text{:FV}^{hm}\text{:FXa}^m] - k_\alpha^-[\text{C:TFPI}\alpha\text{:FV}^{hm}\text{:FXa}^m] \tag{39}$$

4 Supplementary Figures & Tables

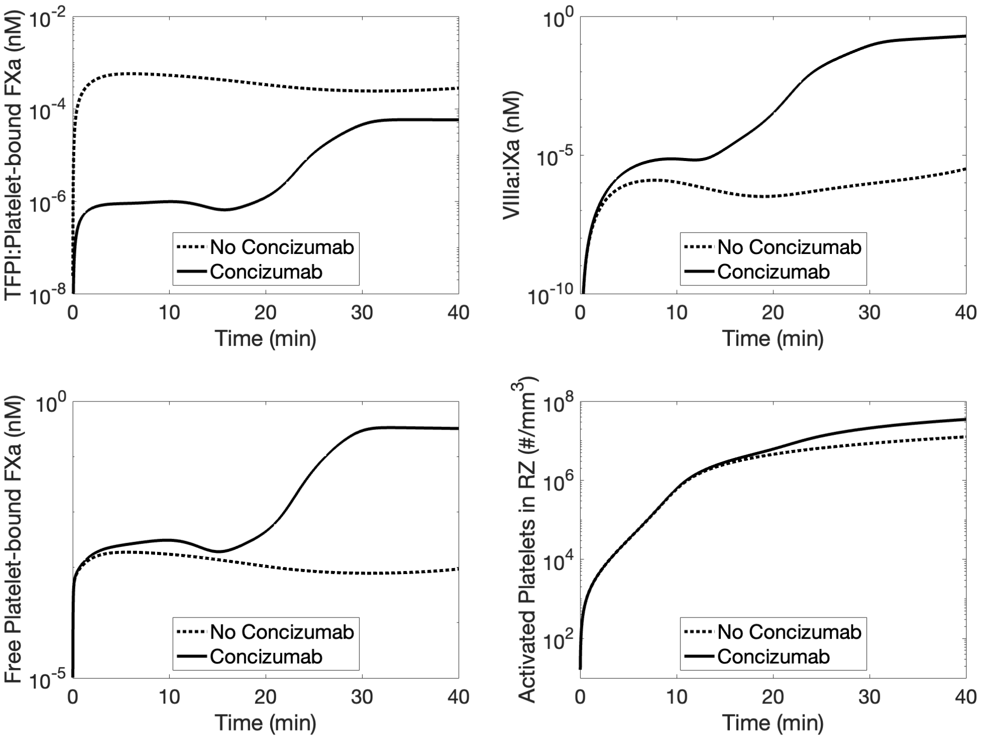

Figure S2: Concentration time courses of selected model species during simulated, flow-mediated coagulation. The panels show FXa:TFPIα bound to the platelet via FXa (top left), platelet-bound tenase (FVIIIa:FIXa) (top right), platelet-bound FXa (bottom left), total activated platelets in the reaction zone (bottom right). Total intravascular concizumab level was 21.5 nM (4.14 nM free plasma concizumab plus 17.36 nM other complexes with TFPIα and TFPIβ). Tissue factor was fixed at 9 fmol/cm^2 and FVIII was 1% (0.01 nM). Concizumab prevented plt-FXa inhibition by TFPIα, enhanced the concentrations of tenase formation, increased the concentration of free plt-FXa, and ultimately increased the total amount of activated platelets in the reaction zone (indirectly by enhancing thrombin generation).

Model Species	Concentration	Note
Prothrombin	1400 nM	a
Factor V	10 nM	b
Factor VII	10 nM	a
Factor VIIa	0.1 nM	c
Factor VIII	1.0 nM	a
Factor IX	90 nM	a
Factor X	170 nM	a
Factor XI	30.0 nM	a
TFPI	0.5 nM	d
Platelet count	$2.5(10)^5/\mu$l	e
N_5	30/plt	*

Table S2: Concentrations of Clotting Factors and Platelets (a) From [10]. (b) From [11]. (c) [12] suggests that normal plasma concentration of FVIIa is about 1% of the normal FVII concentration. (d) From [13]. (e) From [14]. (*) The simulations in this paper use 30 FV molecules per platelet that are released upon platelet activation, however this number is less that what is proposed in the literature [15]. Simulations were also run for higher values up to 3000/platelets and the results did not change qualitatively, but required less TF for initiation of coagulation.

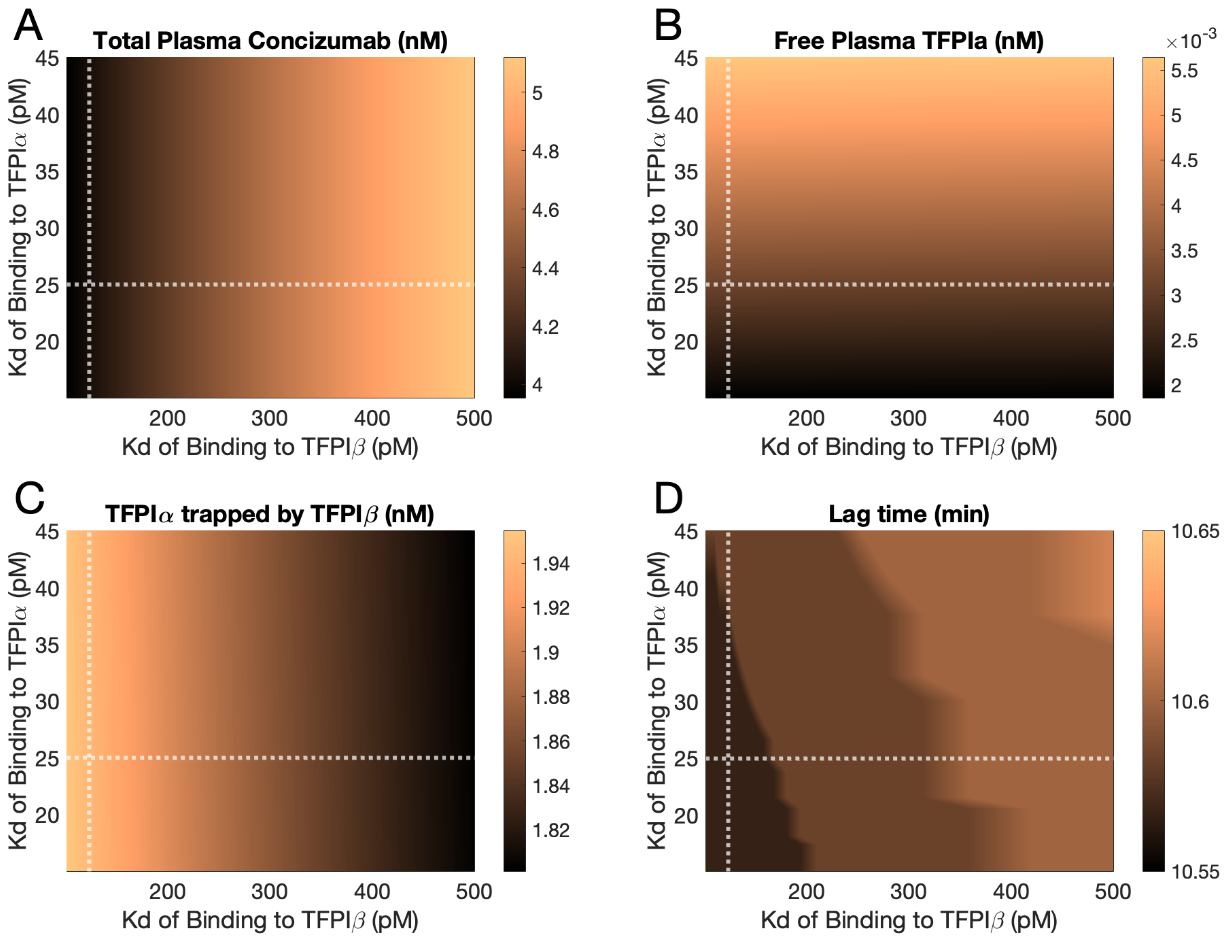

Figure S3: Heat maps of model sensitivity to concizumab dissociation rates. Varying the K_d for concizumab with TFPIβ (along x axis) and with TFPIα (along y axis). The color in the heat maps represent values of the steady state concentrations (A-C) or lag time (D). Outputs are total plasma concizumab (A), free plasma TFPIα (B), TFPIα within in a ternary complex with concizumab and TFPIβ (C), and the resulting lag time when the corresponding steady-state values are used as inputs to the flow-mediated coagulation model (D). For flow model, TF = 9 fmol/cm^2 and FVIII set to 1% (0.01 nM). White dotted lines indicate baseline values used in the simulations in this study.

References

[1] George J Broze Jr and Thomas J Girard. Tissue factor pathway inhibitor: structure-function. *Frontiers in bioscience: a journal and virtual library*, 17:262, 2012.

[2] Julie A Peterson, Susan A Maroney, Nicholas D Martinez, and Alan E Mast. Major reservoir for heparin-releasable tfpiα (tissue factor pathway inhibitor α) is extracellular matrix. *Arteriosclerosis, thrombosis, and vascular biology*, 41(6):1942–1955, 2021.

[3] George J. Broze and Thomas J. Girard. Factor V, tissue factor pathway inhibitor, and east Texas bleeding disorder. *J. Clin. Invest.*, 123(9):3710–3712, September 2013.

[4] Dongfen Yuan, Frederik Rode, and Yanguang Cao. A systems pharmacokinetic/pharmacodynamic model for concizumab to explore the potential of anti-tfpi recycling antibodies. *European Journal of Pharmaceutical Sciences*, 138:105032, 2019.

[5] Andrew L. Kuharsky and Aaron L. Fogelson. Surface-Mediated Control of Blood Coagulation: The Role of Binding Site Densities and Platelet Deposition. *Biophysical Journal*, 80(3):1050–1074, March 2001.

[6] Aaron L. Fogelson and Nessy Tania. Coagulation under Flow: The Influence of Flow-Mediated Transport on the Initiation and Inhibition of Coagulation. *Pathophysiol Haemos Thromb*, 34(2-3):91–108, 2005.

[7] Aaron L. Fogelson, Yasmeen H. Hussain, and Karin Leiderman. Blood Clot Formation under Flow: The Importance of Factor XI Depends Strongly on Platelet Count. *Biophysical Journal*, 102(1):10–18, January 2012.

[8] Kenji Miyazawa, Aaron L Fogelson, and Karin Leiderman. Inhibition of platelet-surface-bound proteins during coagulation under flow I: TFPI. *Biophysical journal*, 122(1):99–113, 2023.

[9] Kenji Miyazawa, Aaron L Fogelson, and Karin Leiderman. Inhibition of platelet-surface-bound proteins during coagulation under flow II: Antithrombin and heparin. *Biophysical Journal*, 122(1):230, 2023.

[10] K.G. Mann, M.E. Nesheim, W.R. Church, P. Haley, and S. Krishnaswamy. Surface-dependent reactions of the vitamin K-dependent enzyme complexes. *Blood*, 76:1–16, 1990.

[11] K.G. Mann, E.G. Bovill, and S. Krishnaswamy. Surface-dependent reactions in the propagation phase of blood coagulation. *Ann. N. Y. Acad. Sci.*, 614:63–75, 1991.

[12] J. H. Morrissey. Tissue Factor modulation of Factor VIIa activity: Use in measuring trace levels of Factor VIIa in plasma,. *Thromb. Haemost.*, 74:185–188, 1995.

[13] W.F. Novotny, S.G. Brown, J.P. Miletich, D.J. Rader, and G.J. Broze. Plasma antigen levels of the lipoprotein-associated coagulation inhibitor in patient samples. *Blood*, 78:387–93, 1991.

[14] H. J. Weiss. Platelet physiology and abnormalities of platelet function (Part 1). *New Engl. J. Med.*, 293:531–541, 1975.

[15] P. N. Walsh. Platelet-coagulant protein interactions. In Robert W. Colman, Jack Hirsh, Victor J. Marder, and Edwin W. Salzman, editors, *Hemostasis and Thrombosis: Basic Principles and Clinical Practice*, pages 629–651. J.B. Lippincott Company, Philadelphia, PA, 3d edition, 1994.
